# *catena*-Poly[[[bis­(3-hy­droxy­adamantane-1-carboxyl­ato-κ*O* ^1^)(3-hy­droxy­adamantane-1-carb­oxy­lic acid-κ*O* ^1^)zinc(II)]-μ_2_-4,4′-bipyridine] monohydrate]

**DOI:** 10.1107/S1600536811016424

**Published:** 2011-05-07

**Authors:** Jin-Bei Shen, Quan-Yin Guan, Xiao-Ju Chen, Guo-Liang Zhao

**Affiliations:** a College of Chemistry and Life Science, Zhejiang Normal University, Jinhua 321004, Zhejiang, People’s Republic of China; b Zhejiang Normal University Xingzhi College, Jinhua, Zhejiang 321004, People’s Republic of China

## Abstract

In the title coordination polymer, {[Zn(C_11_H_15_O_3_)_2_(C_10_H_8_N_2_)(C_11_H_16_O_3_)]·H_2_O}_*n*_, the Zn^II^ ion is five coordinated by two N atoms from two 4,4′-bipyridine (4,4′-bpy) mol­ecules and three O atoms from two 3-hy­droxy­adamantane-1-carboxyl­ate anions (*L*) and one 3-hy­droxy­adamantane-1-carb­oxy­lic acid (H*L*) mol­ecule. The resulting coordination polyhedron is a near regular ZnN_2_O_3_ trigonal bipyramid, with the N atoms in axial sites. The 4,4′-bpy mol­ecules [dihedral angle between the aromatic rings = 17.2 (2)°] act as bridges, connecting the metal ions into an infinite polymeric chain propagating in [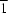
01]. O—H⋯O hydrogen bonds help to consolidate the packing.

## Related literature

For background to adamantane-1-carb­oxy­lic acid complexes, see: Zhu *et al.* (2005 ▶); Milios *et al.* (2007 ▶); Korlyukov *et al.* (2008 ▶).
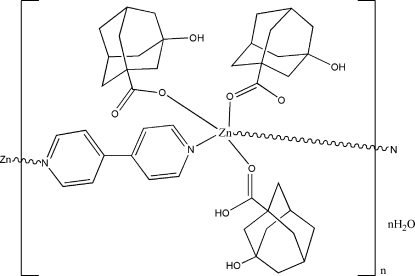


## Experimental

### 

#### Crystal data

[Zn(C_11_H_15_O_3_)_2_(C_10_H_8_N_2_)(C_11_H_16_O_3_)]·H_2_O
*M* *_r_* = 826.27
Monoclinic, 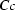

*a* = 17.8778 (2) Å
*b* = 16.6364 (2) Å
*c* = 13.2655 (1) Å
β = 92.642 (1)°
*V* = 3941.26 (7) Å^3^
*Z* = 4
Mo *K*α radiation
μ = 0.69 mm^−1^
*T* = 296 K
0.34 × 0.23 × 0.15 mm

#### Data collection

Bruker APEXII CCD diffractometer
Absorption correction: multi-scan (*SADABS*; Sheldrick, 1996 ▶) *T*_min_ = 0.826, *T*_max_ = 0.904
26431 measured reflections
6904 independent reflections
6188 reflections with *I* > 2σ(*I*)
*R*_int_ = 0.051

#### Refinement

*R*[*F* ^2^ > 2σ(*F* ^2^)] = 0.038
*wR*(*F* ^2^) = 0.094
*S* = 1.04
6904 reflections
506 parameters
4 restraints
H-atom parameters constrained
Δρ_max_ = 0.28 e Å^−3^
Δρ_min_ = −0.33 e Å^−3^
Absolute structure: Flack (1983 ▶), 3423 Friedel pairs
Flack parameter: 0.202 (9)

### 

Data collection: *APEX2* (Bruker, 2006 ▶); cell refinement: *SAINT* (Bruker, 2006 ▶); data reduction: *SAINT*; program(s) used to solve structure: *SHELXS97* (Sheldrick, 2008 ▶); program(s) used to refine structure: *SHELXL97* (Sheldrick, 2008 ▶); molecular graphics: *SHELXTL* (Sheldrick, 2008 ▶); software used to prepare material for publication: *SHELXL97*.

## Supplementary Material

Crystal structure: contains datablocks I, global. DOI: 10.1107/S1600536811016424/hb5859sup1.cif

Structure factors: contains datablocks I. DOI: 10.1107/S1600536811016424/hb5859Isup2.hkl

Additional supplementary materials:  crystallographic information; 3D view; checkCIF report

## Figures and Tables

**Table 1:** Selected bond lengths (Å)

Zn1—O2	2.016 (3)
Zn1—O8	2.036 (3)
Zn1—O4	2.065 (3)
Zn1—N2^i^	2.138 (3)
Zn1—N1	2.174 (4)

Symmetry code: (i) 
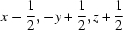
.

**Table 2:** Hydrogen-bond geometry (Å, °)

*D*—H⋯*A*	*D*—H	H⋯*A*	*D*⋯*A*	*D*—H⋯*A*
O9—H9*A*⋯O1^ii^	0.82	2.05	2.872 (4)	178
O6—H6*A*⋯O3^iii^	0.82	2.00	2.812 (4)	169
O3—H3*A*⋯O9^iv^	0.82	1.99	2.790 (4)	165
O1*W*—H1*WA*⋯O6	0.85	2.19	3.013 (9)	163
O5—H5*C*⋯O7	0.85	1.67	2.447 (3)	151
O1*W*—H1*WB*⋯O7^v^	0.85	2.44	3.287 (9)	179

Symmetry codes: (ii) 
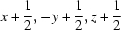
; (iii) 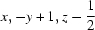; (iv) 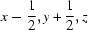; (v) 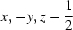.

**Fig. 1.**
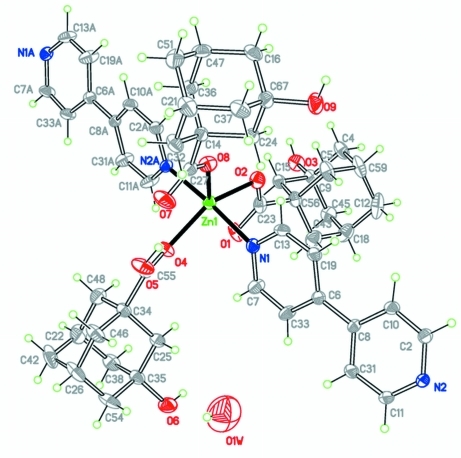
The molecular structure of the title complex, showing displacement ellipsoids drawn at the 30% probability level.

**Fig. 2.**
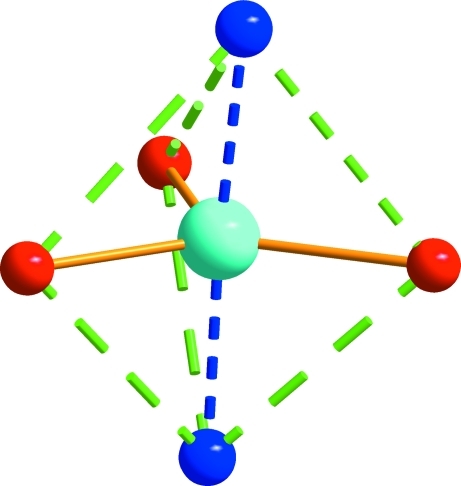
The coordination environment of the ZnII atom, showing the octahedral structurem.

**Fig. 3.**
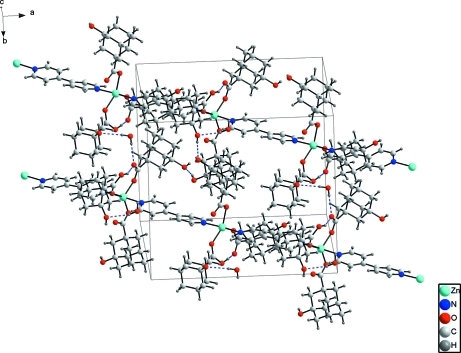
The stacking plot of the title compound, showing H-bond interactions (dashed lines) and π···π stacking interactions.

## supplementary crystallographic information

### Comment

The fascinating structures of adamantane-1- carboxylic acid complexes coupled with their special functionality catch a lot of chemists' interests (Zhu *et al.*,2005; Milios *et al.*,2007; Korlyukov *et al.*,2008). To the best of our knowledge, the polymer complex using 4,4'-bipyridine as the linker and 3-hydroxyadamantane-1- carboxylic acid as filling agent has not been reported up to the present time. As an extension of our work in this field, we describe a new Zn^II^ complex.

The structure of complex (1) was shown in Fig.1 and the coordination environment of Zn^II^ was shown in Fig. 2.In the coordination compound poly, [Zn(C_10_H_8_N_2_).(C_11_H_15_O_3_)_2_.(C_11_H_16_O_3_)]_n_.n(H_2_0), each Zn^II^ ion is five coordinated by two N atoms from two 4,4'-bpy molecules and three O atoms from two 3-hydroxyadamantane-1-carboxylic anions (*L*) and one 3-hydroxy-1-adamantanecarboxylic acid (H*L*). The 4,4'-bpy molecules act as bidentate bridges,connecting the Zn^II^ ions centres in a distorted trigonal-bipyramidal geometry into an infinite polymeric chain.The coordination geometry around Zn^II^ seems to be classified as a trigonal bipyramid; O2, O4 and O8 atoms form the equatorial trigonal plane indicated by the angle of O2—Zn1—O4, O2—Zn1—O8 and O8—Zn1—O4 being 140.19 (11)°,102.42 (11)° and 117.37 (8)°, respectively. The axial position occupy N1 and N2 atoms; N1—Zn—N2 is the only combination with bonding angle close to 180 degrees. The Zn—O and Zn—N bond distances are listed in Table1.

The hydrogen bonds and π···π weak non-covalent interactions lend stability to the structure. The hydrogen bonds are listed in Table 2 and the stacking plot of this compound is shown in Fig.3.

### Experimental

Reagents and solvents used were of commercially available quality and without purified before using. A mixture of 3-hydroxyadamantane-1-carboxylic acid (0.3924 g, 2 mmol), Zn(OH)_2_ (0.0994 g, 1 mmol), 4,4'-bipyridine (0.1562 g, 1 mmol) and water (16 ml) was sealed in a 25 ml stainless steel reactor and heated at 160 K for 2 d and then cooled to room temperature over 3 d. The resulting colourless crystals were obtained and collected by filtration, washed with water, and dried in air (yield 33%).

### Refinement

The structure was solved by direct methods and successive Fourier difference synthesis. The H atoms bonded to C atoms were positioned geometrically and refined using a riding model [aliphatic C—H =0.96 Å (*U*_iso_(H) = 1.5*U*_eq_(C)), aromatic C—H = 0.93 Å (*U*_iso_(H) = 1.2*U*_eq_(C))]. H atoms bonded to O atoms were located in difference Fourier maps and refined with O—H distance restraints of 0.83 (2) and *U*_iso_(H) = 1.5*U*_eq_(O).

### Crystal data

**Table:**

[Zn(C_11_H_15_O_3_)_2_(C_10_H_8_N_2_)(C_11_H_16_O_3_)]·H_2_O	*F*(000) = 1752
*M**_r_* = 826.27	*D*_x_ = 1.392 Mg m^−^^3^
Monoclinic, *C**c*	Mo *K*α radiation, λ = 0.71073 Å
Hall symbol: C -2yc	Cell parameters from 5047 reflections
*a* = 17.8778 (2) Å	θ = 2.3–25.0°
*b* = 16.6364 (2) Å	µ = 0.69 mm^−^^1^
*c* = 13.2655 (1) Å	*T* = 296 K
β = 92.642 (1)°	Block, colourless
*V* = 3941.26 (7) Å^3^	0.34 × 0.23 × 0.15 mm
*Z* = 4	

### Data collection

**Table:**

Bruker APEXII CCD diffractometer	6904 independent reflections
Radiation source: fine-focus sealed tube	6188 reflections with *I* > 2σ(*I*)
graphite	*R*_int_ = 0.051
φ and ω scans	θ_max_ = 25.0°, θ_min_ = 2.3°
Absorption correction: multi-scan (*SADABS*; Sheldrick, 1996)	*h* = −21→21
*T*_min_ = 0.826, *T*_max_ = 0.904	*k* = −18→19
26431 measured reflections	*l* = −15→15

### Refinement

**Table:**

Refinement on *F*^2^	Secondary atom site location: difference Fourier map
Least-squares matrix: full	Hydrogen site location: inferred from neighbouring sites
*R*[*F*^2^ > 2σ(*F*^2^)] = 0.038	H-atom parameters constrained
*wR*(*F*^2^) = 0.094	*w* = 1/[σ^2^(*F*_o_^2^) + (0.0484*P*)^2^ + 1.9133*P*] where *P* = (*F*_o_^2^ + 2*F*_c_^2^)/3
*S* = 1.04	(Δ/σ)_max_ = 0.010
6904 reflections	Δρ_max_ = 0.28 e Å^−^^3^
506 parameters	Δρ_min_ = −0.33 e Å^−^^3^
4 restraints	Absolute structure: Flack (1983), 3423 Friedel pairs
Primary atom site location: structure-invariant direct methods	Flack parameter: 0.202 (9)

### Special details

**Table:**

Geometry. All e.s.d.'s (except the e.s.d. in the dihedral angle between two l.s. planes) are estimated using the full covariance matrix. The cell e.s.d.'s are taken into account individually in the estimation of e.s.d.'s in distances, angles and torsion angles; correlations between e.s.d.'s in cell parameters are only used when they are defined by crystal symmetry. An approximate (isotropic) treatment of cell e.s.d.'s is used for estimating e.s.d.'s involving l.s. planes.
Refinement. Refinement of *F*^2^ against ALL reflections. The weighted *R*-factor *wR* and goodness of fit *S* are based on *F*^2^, conventional *R*-factors *R* are based on *F*, with *F* set to zero for negative *F*^2^. The threshold expression of *F*^2^ > σ(*F*^2^) is used only for calculating *R*-factors(gt) *etc*. and is not relevant to the choice of reflections for refinement. *R*-factors based on *F*^2^ are statistically about twice as large as those based on *F*, and *R*- factors based on ALL data will be even larger.

### Fractional atomic coordinates and isotropic or equivalent isotropic displacement parameters (Å^2^)

**Table:**

	*x*	*y*	*z*	*U*_iso_*/*U*_eq_	
Zn1	0.40049 (2)	0.236430 (19)	0.71677 (3)	0.03051 (11)	
N1	0.49072 (19)	0.23781 (19)	0.6124 (3)	0.0342 (8)	
O4	0.33507 (14)	0.17132 (17)	0.6136 (2)	0.0402 (7)	
O1	0.34888 (16)	0.35868 (16)	0.6198 (2)	0.0550 (7)	
C6	0.6136 (2)	0.2535 (2)	0.4895 (3)	0.0315 (9)	
C7	0.4819 (2)	0.2425 (3)	0.5126 (3)	0.0451 (11)	
H7A	0.4336	0.2408	0.4836	0.054*	
C8	0.6799 (2)	0.2591 (2)	0.4261 (3)	0.0284 (9)	
C9	0.3480 (2)	0.6166 (2)	0.7172 (3)	0.0366 (8)	
C10	0.7489 (2)	0.2815 (2)	0.4668 (3)	0.0356 (9)	
H10A	0.7546	0.2954	0.5346	0.043*	
C13	0.5609 (3)	0.2407 (3)	0.6506 (3)	0.0468 (12)	
H13A	0.5689	0.2376	0.7203	0.056*	
C14	0.54545 (18)	0.0765 (2)	0.9012 (2)	0.0318 (7)	
C15	0.3374 (2)	0.5255 (2)	0.7198 (3)	0.0369 (9)	
H15A	0.2933	0.5109	0.6784	0.044*	
H15B	0.3296	0.5084	0.7884	0.044*	
C16	0.6481 (2)	0.1194 (3)	1.0717 (3)	0.0467 (10)	
H16A	0.6369	0.1762	1.0771	0.056*	
H16B	0.6859	0.1058	1.1236	0.056*	
O9	0.74338 (14)	0.14821 (19)	0.9501 (2)	0.0595 (8)	
H9A	0.7727	0.1453	0.9993	0.089*	
O2	0.42651 (15)	0.35127 (16)	0.7520 (2)	0.0451 (6)	
C19	0.6213 (3)	0.2479 (3)	0.5933 (4)	0.0510 (13)	
H19A	0.6689	0.2490	0.6245	0.061*	
O5	0.38659 (15)	0.05103 (16)	0.6003 (2)	0.0524 (7)	
H5C	0.4219	0.0611	0.6435	0.079*	
C23	0.3919 (2)	0.3914 (2)	0.6833 (3)	0.0413 (9)	
C24	0.61765 (18)	0.1252 (2)	0.8871 (3)	0.0360 (8)	
H24A	0.6361	0.1148	0.8207	0.043*	
H24B	0.6069	0.1822	0.8920	0.043*	
C25	0.3097 (2)	0.1414 (2)	0.3941 (3)	0.0370 (8)	
H25A	0.3058	0.1972	0.4147	0.044*	
H25B	0.3611	0.1312	0.3776	0.044*	
C26	0.2442 (3)	−0.0157 (2)	0.3534 (4)	0.0623 (14)	
H26A	0.2491	−0.0718	0.3320	0.075*	
O8	0.46501 (15)	0.17339 (19)	0.8196 (2)	0.0432 (7)	
C31	0.6747 (3)	0.2418 (3)	0.3243 (4)	0.0467 (12)	
H31A	0.6290	0.2272	0.2933	0.056*	
C32	0.5637 (2)	−0.0133 (2)	0.8930 (3)	0.0448 (9)	
H32A	0.5188	−0.0448	0.9022	0.054*	
H32B	0.5813	−0.0250	0.8265	0.054*	
C33	0.5409 (2)	0.2496 (3)	0.4499 (3)	0.0440 (10)	
H33A	0.5316	0.2518	0.3804	0.053*	
C34	0.28800 (19)	0.0859 (2)	0.4816 (3)	0.0345 (8)	
C35	0.2579 (2)	0.1262 (2)	0.3016 (3)	0.0410 (8)	
C36	0.5172 (2)	0.0942 (3)	1.0063 (3)	0.0430 (9)	
H36A	0.5059	0.1510	1.0121	0.052*	
H36B	0.4716	0.0642	1.0163	0.052*	
C37	0.6940 (2)	0.0121 (2)	0.9582 (3)	0.0459 (9)	
H37A	0.7120	0.0012	0.8917	0.055*	
H37B	0.7328	−0.0034	1.0079	0.055*	
C38	0.1781 (2)	0.1421 (2)	0.3269 (3)	0.0497 (10)	
H38A	0.1453	0.1331	0.2677	0.060*	
H38B	0.1727	0.1977	0.3474	0.060*	
C42	0.1637 (3)	−0.0001 (3)	0.3796 (4)	0.0777 (17)	
H42A	0.1305	−0.0107	0.3213	0.093*	
H42B	0.1501	−0.0357	0.4337	0.093*	
C43	0.4178 (2)	0.5102 (3)	0.5727 (3)	0.0538 (10)	
H43A	0.3749	0.4949	0.5295	0.065*	
H43B	0.4618	0.4842	0.5474	0.065*	
C45	0.3601 (2)	0.6431 (2)	0.6105 (3)	0.0499 (10)	
H45A	0.3673	0.7008	0.6089	0.060*	
H45B	0.3160	0.6303	0.5680	0.060*	
C46	0.2954 (3)	−0.0014 (2)	0.4472 (3)	0.0524 (11)	
H46A	0.3470	−0.0124	0.4319	0.063*	
H46B	0.2817	−0.0373	0.5009	0.063*	
C47	0.5774 (2)	0.0703 (3)	1.0867 (3)	0.0518 (11)	
H47A	0.5591	0.0811	1.1540	0.062*	
C48	0.2070 (2)	0.1033 (3)	0.5065 (3)	0.0502 (10)	
H48A	0.2021	0.1590	0.5269	0.060*	
H48B	0.1927	0.0694	0.5619	0.060*	
C51	0.5954 (3)	−0.0193 (3)	1.0770 (3)	0.0564 (12)	
H51A	0.6330	−0.0346	1.1285	0.068*	
H51B	0.5507	−0.0508	1.0869	0.068*	
C54	0.2659 (3)	0.0384 (2)	0.2694 (3)	0.0588 (12)	
H54A	0.2339	0.0281	0.2097	0.071*	
H54B	0.3172	0.0278	0.2529	0.071*	
C55	0.34004 (18)	0.1055 (2)	0.5728 (2)	0.0310 (7)	
C56	0.40623 (18)	0.4824 (2)	0.6804 (3)	0.0373 (8)	
O6	0.28053 (17)	0.17504 (18)	0.21918 (19)	0.0573 (7)	
H6A	0.2791	0.2226	0.2353	0.086*	
C59	0.4852 (2)	0.5973 (2)	0.7440 (4)	0.0578 (13)	
H59A	0.5291	0.6120	0.7871	0.069*	
C67	0.67741 (19)	0.1016 (2)	0.9682 (3)	0.0409 (9)	
C4	0.4164 (2)	0.6388 (2)	0.7831 (3)	0.0504 (11)	
H4A	0.4092	0.6224	0.8521	0.060*	
H4B	0.4235	0.6966	0.7822	0.060*	
C5	0.4746 (2)	0.5069 (3)	0.7476 (4)	0.0523 (11)	
H5A	0.4675	0.4901	0.8166	0.063*	
H5B	0.5190	0.4804	0.7245	0.063*	
C18	0.4279 (3)	0.6016 (3)	0.5697 (4)	0.0606 (12)	
H18A	0.4347	0.6191	0.5002	0.073*	
C21	0.6240 (2)	−0.0364 (2)	0.9738 (3)	0.0519 (10)	
H21A	0.6355	−0.0938	0.9681	0.062*	
C12	0.4973 (3)	0.6246 (3)	0.6368 (5)	0.0763 (17)	
H12A	0.5415	0.5990	0.6119	0.092*	
H12B	0.5047	0.6824	0.6353	0.092*	
C22	0.1556 (2)	0.0865 (3)	0.4123 (4)	0.0629 (13)	
H22A	0.1035	0.0969	0.4279	0.075*	
N2	0.80564 (18)	0.26537 (18)	0.3092 (2)	0.0287 (7)	
C11	0.7389 (3)	0.2465 (3)	0.2685 (4)	0.0477 (12)	
H11A	0.7347	0.2358	0.1996	0.057*	
O3	0.28231 (15)	0.65761 (16)	0.7492 (2)	0.0498 (7)	
H3A	0.2777	0.6498	0.8096	0.075*	
C27	0.48627 (19)	0.1019 (2)	0.8201 (3)	0.0324 (8)	
O7	0.46364 (17)	0.04954 (18)	0.7576 (2)	0.0600 (8)	
C2	0.8096 (2)	0.2832 (2)	0.4060 (3)	0.0346 (9)	
H2A	0.8560	0.2977	0.4351	0.041*	
O1W	0.4470 (4)	0.1457 (5)	0.2261 (7)	0.208 (3)	
H1WA	0.4019	0.1602	0.2137	0.312*	
H1WB	0.4517	0.0953	0.2344	0.312*	

### Atomic displacement parameters (Å^2^)

**Table:**

	*U*^11^	*U*^22^	*U*^33^	*U*^12^	*U*^13^	*U*^23^
Zn1	0.02855 (17)	0.03075 (18)	0.03260 (18)	0.0000 (2)	0.00535 (12)	−0.0010 (2)
N1	0.0286 (19)	0.0376 (19)	0.0367 (19)	−0.0029 (14)	0.0051 (11)	−0.0037 (14)
O4	0.0350 (15)	0.0427 (17)	0.0420 (15)	0.0039 (13)	−0.0075 (12)	−0.0146 (13)
O1	0.0441 (16)	0.0421 (16)	0.078 (2)	−0.0102 (13)	−0.0111 (14)	−0.0143 (15)
C6	0.038 (2)	0.0271 (19)	0.030 (2)	−0.0022 (15)	0.0033 (17)	−0.0004 (15)
C7	0.028 (2)	0.076 (3)	0.031 (2)	0.0024 (18)	−0.0029 (16)	0.0126 (19)
C8	0.026 (2)	0.032 (2)	0.028 (2)	0.0012 (15)	0.0053 (16)	0.0020 (14)
C9	0.0324 (19)	0.032 (2)	0.045 (2)	0.0044 (15)	−0.0072 (16)	−0.0033 (16)
C10	0.032 (2)	0.049 (2)	0.0253 (18)	0.0004 (17)	−0.0025 (15)	0.0017 (16)
C13	0.040 (3)	0.076 (3)	0.024 (2)	−0.013 (2)	0.0011 (18)	−0.0085 (19)
C14	0.0260 (17)	0.0367 (19)	0.0321 (17)	−0.0032 (14)	−0.0049 (14)	0.0056 (14)
C15	0.0301 (19)	0.040 (2)	0.040 (2)	0.0002 (15)	−0.0053 (15)	0.0010 (16)
C16	0.045 (2)	0.052 (3)	0.041 (2)	−0.0021 (19)	−0.0161 (18)	−0.0019 (19)
O9	0.0358 (15)	0.079 (2)	0.0625 (18)	−0.0204 (14)	−0.0140 (13)	0.0203 (15)
O2	0.0511 (17)	0.0302 (14)	0.0541 (17)	0.0002 (12)	0.0042 (13)	0.0011 (13)
C19	0.029 (2)	0.090 (4)	0.034 (2)	−0.021 (2)	0.0011 (19)	−0.012 (2)
O5	0.0559 (16)	0.0543 (17)	0.0440 (15)	0.0203 (13)	−0.0290 (12)	−0.0091 (13)
C23	0.0284 (18)	0.037 (2)	0.059 (3)	0.0026 (17)	0.0120 (18)	−0.0052 (18)
C24	0.0297 (18)	0.042 (2)	0.0355 (19)	−0.0047 (15)	−0.0045 (14)	0.0057 (16)
C25	0.039 (2)	0.038 (2)	0.0338 (18)	0.0016 (16)	−0.0054 (15)	−0.0026 (15)
C26	0.088 (4)	0.029 (2)	0.065 (3)	−0.002 (2)	−0.046 (3)	−0.006 (2)
O8	0.0407 (17)	0.049 (2)	0.0394 (15)	0.0108 (13)	0.0000 (11)	0.0060 (12)
C31	0.028 (2)	0.077 (3)	0.034 (2)	−0.015 (2)	0.0000 (18)	−0.013 (2)
C32	0.050 (2)	0.033 (2)	0.049 (2)	−0.0037 (16)	−0.0169 (18)	0.0032 (16)
C33	0.028 (2)	0.076 (3)	0.0282 (19)	0.0044 (18)	0.0003 (15)	0.0115 (18)
C34	0.0327 (18)	0.035 (2)	0.0344 (19)	0.0031 (15)	−0.0096 (15)	−0.0009 (16)
C35	0.050 (2)	0.037 (2)	0.0350 (19)	−0.0001 (17)	−0.0116 (16)	0.0002 (15)
C36	0.0321 (19)	0.063 (3)	0.034 (2)	−0.0002 (18)	−0.0014 (16)	0.0060 (19)
C37	0.040 (2)	0.051 (2)	0.045 (2)	0.0116 (17)	−0.0165 (17)	0.0024 (18)
C38	0.050 (2)	0.047 (2)	0.050 (2)	0.0072 (19)	−0.0202 (19)	0.0004 (19)
C42	0.082 (4)	0.065 (3)	0.081 (3)	−0.039 (3)	−0.051 (3)	0.022 (3)
C43	0.055 (2)	0.053 (3)	0.055 (2)	0.004 (2)	0.017 (2)	−0.003 (2)
C45	0.058 (3)	0.039 (2)	0.052 (2)	0.0048 (19)	−0.0014 (19)	0.0067 (19)
C46	0.071 (3)	0.030 (2)	0.053 (2)	0.0039 (18)	−0.034 (2)	0.0004 (17)
C47	0.047 (2)	0.082 (3)	0.0253 (18)	−0.002 (2)	−0.0054 (16)	0.0063 (19)
C48	0.036 (2)	0.071 (3)	0.043 (2)	−0.006 (2)	−0.0040 (17)	0.013 (2)
C51	0.049 (2)	0.065 (3)	0.054 (3)	−0.007 (2)	−0.017 (2)	0.030 (2)
C54	0.081 (3)	0.047 (2)	0.045 (2)	0.014 (2)	−0.032 (2)	−0.0174 (19)
C55	0.0309 (18)	0.039 (2)	0.0232 (17)	−0.0029 (15)	0.0012 (14)	0.0022 (15)
C56	0.0292 (19)	0.0337 (19)	0.049 (2)	0.0010 (14)	−0.0008 (16)	−0.0007 (15)
O6	0.080 (2)	0.0559 (17)	0.0351 (14)	−0.0023 (15)	−0.0072 (13)	0.0070 (13)
C59	0.034 (2)	0.034 (2)	0.104 (4)	−0.0015 (18)	−0.018 (2)	−0.008 (2)
C67	0.0300 (18)	0.050 (2)	0.042 (2)	−0.0058 (16)	−0.0042 (16)	0.0049 (18)
C4	0.049 (2)	0.031 (2)	0.069 (3)	0.0017 (18)	−0.019 (2)	−0.003 (2)
C5	0.033 (2)	0.040 (2)	0.083 (3)	0.0045 (17)	−0.013 (2)	−0.005 (2)
C18	0.072 (3)	0.044 (2)	0.068 (3)	0.003 (2)	0.025 (2)	0.012 (2)
C21	0.058 (3)	0.036 (2)	0.059 (3)	0.0014 (19)	−0.022 (2)	0.0099 (19)
C12	0.048 (3)	0.044 (3)	0.139 (5)	−0.008 (2)	0.026 (3)	0.014 (3)
C22	0.034 (2)	0.089 (4)	0.063 (3)	−0.014 (2)	−0.018 (2)	0.014 (3)
N2	0.0252 (18)	0.0339 (18)	0.0274 (17)	−0.0012 (13)	0.0046 (12)	−0.0020 (13)
C11	0.038 (3)	0.077 (3)	0.029 (2)	−0.016 (2)	0.0085 (19)	−0.0156 (19)
O3	0.0445 (15)	0.0465 (16)	0.0580 (17)	0.0159 (13)	0.0004 (12)	−0.0042 (13)
C27	0.0278 (18)	0.040 (2)	0.0298 (18)	0.0000 (15)	0.0022 (14)	0.0055 (15)
O7	0.0702 (19)	0.0553 (18)	0.0512 (17)	0.0053 (15)	−0.0338 (15)	−0.0030 (14)
C2	0.0252 (18)	0.048 (2)	0.0304 (19)	−0.0002 (16)	−0.0010 (15)	0.0030 (17)
O1W	0.178 (6)	0.206 (8)	0.245 (9)	0.019 (5)	0.065 (6)	−0.016 (6)

### Geometric parameters (Å, °)

**Table:**

Zn1—O2	2.016 (3)	C34—C46	1.530 (5)
Zn1—O8	2.036 (3)	C35—O6	1.435 (4)
Zn1—O4	2.065 (3)	C35—C38	1.504 (5)
Zn1—N2^i^	2.138 (3)	C35—C54	1.531 (5)
Zn1—N1	2.174 (4)	C36—C47	1.533 (5)
N1—C7	1.329 (6)	C36—H36A	0.9700
N1—C13	1.332 (6)	C36—H36B	0.9700
O4—C55	1.227 (4)	C37—C21	1.510 (6)
O1—C23	1.240 (5)	C37—C67	1.525 (5)
C6—C19	1.381 (7)	C37—H37A	0.9700
C6—C33	1.381 (6)	C37—H37B	0.9700
C6—C8	1.489 (4)	C38—C22	1.531 (6)
C7—C33	1.377 (6)	C38—H38A	0.9700
C7—H7A	0.9300	C38—H38B	0.9700
C8—C10	1.374 (6)	C42—C22	1.513 (8)
C8—C31	1.379 (6)	C42—H42A	0.9700
C9—O3	1.438 (4)	C42—H42B	0.9700
C9—C45	1.508 (6)	C43—C56	1.525 (5)
C9—C4	1.515 (5)	C43—C18	1.533 (6)
C9—C15	1.529 (5)	C43—H43A	0.9700
C10—C2	1.383 (5)	C43—H43B	0.9700
C10—H10A	0.9300	C45—C18	1.516 (6)
C13—C19	1.353 (7)	C45—H45A	0.9700
C13—H13A	0.9300	C45—H45B	0.9700
C14—C27	1.533 (4)	C46—H46A	0.9700
C14—C36	1.534 (5)	C46—H46B	0.9700
C14—C32	1.534 (5)	C47—C51	1.532 (7)
C14—C24	1.543 (4)	C47—H47A	0.9800
C15—C56	1.537 (5)	C48—C22	1.543 (6)
C15—H15A	0.9700	C48—H48A	0.9700
C15—H15B	0.9700	C48—H48B	0.9700
C16—C67	1.521 (5)	C51—C21	1.511 (6)
C16—C47	1.525 (6)	C51—H51A	0.9700
C16—H16A	0.9700	C51—H51B	0.9700
C16—H16B	0.9700	C54—H54A	0.9700
O9—C67	1.441 (4)	C54—H54B	0.9700
O9—H9A	0.8200	C56—C5	1.535 (5)
O2—C23	1.267 (4)	O6—H6A	0.8200
C19—H19A	0.9300	C59—C5	1.517 (6)
O5—C55	1.272 (4)	C59—C12	1.518 (7)
O5—H5C	0.8499	C59—C4	1.522 (6)
C23—C56	1.536 (5)	C59—H59A	0.9800
C24—C67	1.532 (5)	C4—H4A	0.9700
C24—H24A	0.9700	C4—H4B	0.9700
C24—H24B	0.9700	C5—H5A	0.9700
C25—C35	1.524 (5)	C5—H5B	0.9700
C25—C34	1.546 (5)	C18—C12	1.541 (7)
C25—H25A	0.9700	C18—H18A	0.9800
C25—H25B	0.9700	C21—H21A	0.9800
C26—C54	1.498 (7)	C12—H12A	0.9700
C26—C42	1.518 (8)	C12—H12B	0.9700
C26—C46	1.529 (5)	C22—H22A	0.9800
C26—H26A	0.9800	N2—C2	1.316 (5)
O8—C27	1.248 (5)	N2—C11	1.324 (6)
C31—C11	1.397 (7)	N2—Zn1^ii^	2.138 (3)
C31—H31A	0.9300	C11—H11A	0.9300
C32—C21	1.533 (5)	O3—H3A	0.8200
C32—H32A	0.9700	C27—O7	1.257 (4)
C32—H32B	0.9700	C2—H2A	0.9300
C33—H33A	0.9300	O1W—H1WA	0.8499
C34—C55	1.527 (4)	O1W—H1WB	0.8499
C34—C48	1.527 (5)		
			
O2—Zn1—O8	102.42 (12)	H38A—C38—H38B	108.2
O2—Zn1—O4	140.19 (12)	C22—C42—C26	109.4 (4)
O8—Zn1—O4	117.37 (9)	C22—C42—H42A	109.8
O2—Zn1—N2^i^	93.50 (11)	C26—C42—H42A	109.8
O8—Zn1—N2^i^	92.77 (11)	C22—C42—H42B	109.8
O4—Zn1—N2^i^	86.13 (11)	C26—C42—H42B	109.8
O2—Zn1—N1	88.14 (11)	H42A—C42—H42B	108.2
O8—Zn1—N1	91.03 (12)	C56—C43—C18	110.2 (3)
O4—Zn1—N1	89.88 (12)	C56—C43—H43A	109.6
N2^i^—Zn1—N1	175.44 (16)	C18—C43—H43A	109.6
C7—N1—C13	116.3 (4)	C56—C43—H43B	109.6
C7—N1—Zn1	125.3 (3)	C18—C43—H43B	109.6
C13—N1—Zn1	118.2 (3)	H43A—C43—H43B	108.1
C55—O4—Zn1	135.3 (2)	C9—C45—C18	110.7 (3)
C19—C6—C33	115.1 (4)	C9—C45—H45A	109.5
C19—C6—C8	121.5 (4)	C18—C45—H45A	109.5
C33—C6—C8	123.3 (3)	C9—C45—H45B	109.5
N1—C7—C33	123.3 (4)	C18—C45—H45B	109.5
N1—C7—H7A	118.4	H45A—C45—H45B	108.1
C33—C7—H7A	118.4	C26—C46—C34	109.5 (3)
C10—C8—C31	117.4 (4)	C26—C46—H46A	109.8
C10—C8—C6	121.2 (3)	C34—C46—H46A	109.8
C31—C8—C6	121.4 (3)	C26—C46—H46B	109.8
O3—C9—C45	107.0 (3)	C34—C46—H46B	109.8
O3—C9—C4	111.3 (3)	H46A—C46—H46B	108.2
C45—C9—C4	108.9 (4)	C16—C47—C51	109.3 (4)
O3—C9—C15	111.1 (3)	C16—C47—C36	109.2 (3)
C45—C9—C15	109.5 (3)	C51—C47—C36	109.7 (3)
C4—C9—C15	109.1 (3)	C16—C47—H47A	109.5
C8—C10—C2	119.4 (3)	C51—C47—H47A	109.5
C8—C10—H10A	120.3	C36—C47—H47A	109.5
C2—C10—H10A	120.3	C34—C48—C22	109.0 (4)
N1—C13—C19	123.4 (5)	C34—C48—H48A	109.9
N1—C13—H13A	118.3	C22—C48—H48A	109.9
C19—C13—H13A	118.3	C34—C48—H48B	109.9
C27—C14—C36	109.8 (3)	C22—C48—H48B	109.9
C27—C14—C32	111.2 (3)	H48A—C48—H48B	108.3
C36—C14—C32	109.4 (3)	C21—C51—C47	110.0 (3)
C27—C14—C24	108.9 (3)	C21—C51—H51A	109.7
C36—C14—C24	108.7 (3)	C47—C51—H51A	109.7
C32—C14—C24	108.8 (3)	C21—C51—H51B	109.7
C9—C15—C56	110.7 (3)	C47—C51—H51B	109.7
C9—C15—H15A	109.5	H51A—C51—H51B	108.2
C56—C15—H15A	109.5	C26—C54—C35	109.6 (3)
C9—C15—H15B	109.5	C26—C54—H54A	109.8
C56—C15—H15B	109.5	C35—C54—H54A	109.8
H15A—C15—H15B	108.1	C26—C54—H54B	109.8
C67—C16—C47	109.7 (3)	C35—C54—H54B	109.8
C67—C16—H16A	109.7	H54A—C54—H54B	108.2
C47—C16—H16A	109.7	O4—C55—O5	124.7 (3)
C67—C16—H16B	109.7	O4—C55—C34	119.1 (3)
C47—C16—H16B	109.7	O5—C55—C34	116.2 (3)
H16A—C16—H16B	108.2	C43—C56—C5	109.0 (3)
C67—O9—H9A	109.5	C43—C56—C23	110.6 (3)
C23—O2—Zn1	103.4 (2)	C5—C56—C23	112.1 (3)
C13—C19—C6	121.4 (4)	C43—C56—C15	108.8 (3)
C13—C19—H19A	119.3	C5—C56—C15	107.9 (3)
C6—C19—H19A	119.3	C23—C56—C15	108.3 (3)
C55—O5—H5C	120.7	C35—O6—H6A	109.5
O1—C23—O2	121.6 (4)	C5—C59—C12	110.5 (4)
O1—C23—C56	120.9 (4)	C5—C59—C4	109.6 (4)
O2—C23—C56	117.4 (3)	C12—C59—C4	109.6 (4)
C67—C24—C14	109.9 (3)	C5—C59—H59A	109.1
C67—C24—H24A	109.7	C12—C59—H59A	109.1
C14—C24—H24A	109.7	C4—C59—H59A	109.1
C67—C24—H24B	109.7	O9—C67—C16	111.4 (3)
C14—C24—H24B	109.7	O9—C67—C37	110.3 (3)
H24A—C24—H24B	108.2	C16—C67—C37	110.2 (3)
C35—C25—C34	109.9 (3)	O9—C67—C24	107.1 (3)
C35—C25—H25A	109.7	C16—C67—C24	109.0 (3)
C34—C25—H25A	109.7	C37—C67—C24	108.7 (3)
C35—C25—H25B	109.7	C9—C4—C59	109.7 (3)
C34—C25—H25B	109.7	C9—C4—H4A	109.7
H25A—C25—H25B	108.2	C59—C4—H4A	109.7
C54—C26—C42	110.3 (4)	C9—C4—H4B	109.7
C54—C26—C46	110.3 (4)	C59—C4—H4B	109.7
C42—C26—C46	109.0 (4)	H4A—C4—H4B	108.2
C54—C26—H26A	109.1	C59—C5—C56	110.0 (3)
C42—C26—H26A	109.1	C59—C5—H5A	109.7
C46—C26—H26A	109.1	C56—C5—H5A	109.7
C27—O8—Zn1	131.1 (3)	C59—C5—H5B	109.7
C8—C31—C11	119.2 (4)	C56—C5—H5B	109.7
C8—C31—H31A	120.4	H5A—C5—H5B	108.2
C11—C31—H31A	120.4	C45—C18—C43	110.2 (3)
C21—C32—C14	109.8 (3)	C45—C18—C12	108.5 (4)
C21—C32—H32A	109.7	C43—C18—C12	108.9 (4)
C14—C32—H32A	109.7	C45—C18—H18A	109.7
C21—C32—H32B	109.7	C43—C18—H18A	109.7
C14—C32—H32B	109.7	C12—C18—H18A	109.7
H32A—C32—H32B	108.2	C37—C21—C51	109.9 (3)
C7—C33—C6	120.5 (4)	C37—C21—C32	109.3 (3)
C7—C33—H33A	119.8	C51—C21—C32	109.2 (4)
C6—C33—H33A	119.8	C37—C21—H21A	109.5
C55—C34—C48	109.7 (3)	C51—C21—H21A	109.5
C55—C34—C46	112.3 (3)	C32—C21—H21A	109.5
C48—C34—C46	109.9 (3)	C59—C12—C18	108.8 (4)
C55—C34—C25	107.6 (3)	C59—C12—H12A	109.9
C48—C34—C25	108.8 (3)	C18—C12—H12A	109.9
C46—C34—C25	108.4 (3)	C59—C12—H12B	109.9
O6—C35—C38	112.0 (3)	C18—C12—H12B	109.9
O6—C35—C25	109.7 (3)	H12A—C12—H12B	108.3
C38—C35—C25	109.8 (3)	C42—C22—C38	109.4 (4)
O6—C35—C54	107.1 (3)	C42—C22—C48	110.1 (4)
C38—C35—C54	109.4 (3)	C38—C22—C48	108.9 (4)
C25—C35—C54	108.8 (3)	C42—C22—H22A	109.5
C47—C36—C14	109.4 (3)	C38—C22—H22A	109.5
C47—C36—H36A	109.8	C48—C22—H22A	109.5
C14—C36—H36A	109.8	C2—N2—C11	117.3 (4)
C47—C36—H36B	109.8	C2—N2—Zn1^ii^	123.6 (3)
C14—C36—H36B	109.8	C11—N2—Zn1^ii^	119.1 (3)
H36A—C36—H36B	108.2	N2—C11—C31	122.9 (4)
C21—C37—C67	110.1 (3)	N2—C11—H11A	118.5
C21—C37—H37A	109.6	C31—C11—H11A	118.5
C67—C37—H37A	109.6	C9—O3—H3A	109.5
C21—C37—H37B	109.6	O8—C27—O7	124.7 (3)
C67—C37—H37B	109.6	O8—C27—C14	117.8 (3)
H37A—C37—H37B	108.1	O7—C27—C14	117.5 (3)
C35—C38—C22	110.0 (3)	N2—C2—C10	123.7 (4)
C35—C38—H38A	109.7	N2—C2—H2A	118.1
C22—C38—H38A	109.7	C10—C2—H2A	118.1
C35—C38—H38B	109.7	H1WA—O1W—H1WB	113.0
C22—C38—H38B	109.7		
			
O2—Zn1—N1—C7	−104.9 (3)	C42—C26—C54—C35	59.7 (4)
O8—Zn1—N1—C7	152.7 (3)	C46—C26—C54—C35	−60.7 (5)
O4—Zn1—N1—C7	35.3 (3)	O6—C35—C54—C26	179.0 (3)
O2—Zn1—N1—C13	69.4 (3)	C38—C35—C54—C26	−59.5 (4)
O8—Zn1—N1—C13	−33.0 (3)	C25—C35—C54—C26	60.5 (4)
O4—Zn1—N1—C13	−150.4 (3)	Zn1—O4—C55—O5	13.2 (6)
O2—Zn1—O4—C55	148.0 (3)	Zn1—O4—C55—C34	−166.1 (3)
O8—Zn1—O4—C55	−30.1 (4)	C48—C34—C55—O4	−51.1 (4)
N2^i^—Zn1—O4—C55	−121.2 (4)	C46—C34—C55—O4	−173.6 (3)
N1—Zn1—O4—C55	61.0 (3)	C25—C34—C55—O4	67.1 (4)
C13—N1—C7—C33	0.2 (6)	C48—C34—C55—O5	129.6 (3)
Zn1—N1—C7—C33	174.7 (3)	C46—C34—C55—O5	7.0 (5)
C19—C6—C8—C10	−18.6 (5)	C25—C34—C55—O5	−112.2 (3)
C33—C6—C8—C10	164.7 (4)	C18—C43—C56—C5	−59.3 (4)
C19—C6—C8—C31	161.2 (5)	C18—C43—C56—C23	177.0 (3)
C33—C6—C8—C31	−15.6 (5)	C18—C43—C56—C15	58.1 (4)
C31—C8—C10—C2	−2.1 (6)	O1—C23—C56—C43	−43.9 (5)
C6—C8—C10—C2	177.7 (3)	O2—C23—C56—C43	135.1 (3)
C7—N1—C13—C19	0.0 (7)	O1—C23—C56—C5	−165.8 (4)
Zn1—N1—C13—C19	−174.9 (4)	O2—C23—C56—C5	13.2 (5)
O3—C9—C15—C56	177.1 (3)	O1—C23—C56—C15	75.2 (4)
C45—C9—C15—C56	59.2 (4)	O2—C23—C56—C15	−105.8 (4)
C4—C9—C15—C56	−59.9 (4)	C9—C15—C56—C43	−58.9 (4)
O8—Zn1—O2—C23	174.5 (2)	C9—C15—C56—C5	59.2 (4)
O4—Zn1—O2—C23	−3.7 (3)	C9—C15—C56—C23	−179.2 (3)
N2^i^—Zn1—O2—C23	−91.9 (2)	C47—C16—C67—O9	178.7 (3)
N1—Zn1—O2—C23	83.9 (2)	C47—C16—C67—C37	−58.5 (4)
N1—C13—C19—C6	0.6 (8)	C47—C16—C67—C24	60.7 (4)
C33—C6—C19—C13	−1.3 (7)	C21—C37—C67—O9	−178.1 (3)
C8—C6—C19—C13	−178.3 (4)	C21—C37—C67—C16	58.5 (4)
Zn1—O2—C23—O1	5.4 (4)	C21—C37—C67—C24	−60.9 (4)
Zn1—O2—C23—C56	−173.6 (2)	C14—C24—C67—O9	179.3 (3)
C27—C14—C24—C67	179.3 (3)	C14—C24—C67—C16	−60.1 (4)
C36—C14—C24—C67	59.6 (4)	C14—C24—C67—C37	60.0 (4)
C32—C14—C24—C67	−59.4 (4)	O3—C9—C4—C59	−177.4 (3)
O2—Zn1—O8—C27	−160.3 (3)	C45—C9—C4—C59	−59.8 (4)
O4—Zn1—O8—C27	18.4 (4)	C15—C9—C4—C59	59.7 (5)
N2^i^—Zn1—O8—C27	105.5 (3)	C5—C59—C4—C9	−60.6 (5)
N1—Zn1—O8—C27	−72.0 (3)	C12—C59—C4—C9	60.7 (5)
C10—C8—C31—C11	1.0 (6)	C12—C59—C5—C56	−60.0 (5)
C6—C8—C31—C11	−178.8 (4)	C4—C59—C5—C56	60.8 (5)
C27—C14—C32—C21	178.9 (3)	C43—C56—C5—C59	58.5 (5)
C36—C14—C32—C21	−59.7 (4)	C23—C56—C5—C59	−178.6 (4)
C24—C14—C32—C21	59.0 (4)	C15—C56—C5—C59	−59.5 (5)
N1—C7—C33—C6	−1.0 (7)	C9—C45—C18—C43	58.6 (5)
C19—C6—C33—C7	1.5 (6)	C9—C45—C18—C12	−60.6 (5)
C8—C6—C33—C7	178.4 (4)	C56—C43—C18—C45	−58.4 (5)
C35—C25—C34—C55	−178.3 (3)	C56—C43—C18—C12	60.5 (4)
C35—C25—C34—C48	−59.5 (4)	C67—C37—C21—C51	−58.9 (4)
C35—C25—C34—C46	59.9 (4)	C67—C37—C21—C32	60.9 (4)
C34—C25—C35—O6	−177.2 (3)	C47—C51—C21—C37	59.7 (4)
C34—C25—C35—C38	59.3 (4)	C47—C51—C21—C32	−60.2 (4)
C34—C25—C35—C54	−60.3 (4)	C14—C32—C21—C37	−60.1 (4)
C27—C14—C36—C47	−178.7 (3)	C14—C32—C21—C51	60.3 (4)
C32—C14—C36—C47	59.0 (4)	C5—C59—C12—C18	60.4 (5)
C24—C14—C36—C47	−59.7 (4)	C4—C59—C12—C18	−60.4 (5)
O6—C35—C38—C22	177.8 (3)	C45—C18—C12—C59	59.9 (5)
C25—C35—C38—C22	−60.1 (4)	C43—C18—C12—C59	−60.1 (5)
C54—C35—C38—C22	59.3 (4)	C26—C42—C22—C38	58.9 (4)
C54—C26—C42—C22	−59.9 (5)	C26—C42—C22—C48	−60.7 (5)
C46—C26—C42—C22	61.3 (5)	C35—C38—C22—C42	−59.5 (4)
O3—C9—C45—C18	−179.1 (3)	C35—C38—C22—C48	60.8 (5)
C4—C9—C45—C18	60.5 (4)	C34—C48—C22—C42	59.0 (5)
C15—C9—C45—C18	−58.7 (4)	C34—C48—C22—C38	−61.0 (5)
C54—C26—C46—C34	60.5 (5)	C2—N2—C11—C31	−2.5 (7)
C42—C26—C46—C34	−60.8 (5)	Zn1^ii^—N2—C11—C31	176.4 (4)
C55—C34—C46—C26	−177.8 (3)	C8—C31—C11—N2	1.4 (8)
C48—C34—C46—C26	59.8 (5)	Zn1—O8—C27—O7	−8.5 (6)
C25—C34—C46—C26	−59.0 (4)	Zn1—O8—C27—C14	170.1 (2)
C67—C16—C47—C51	58.8 (4)	C36—C14—C27—O8	57.2 (4)
C67—C16—C47—C36	−61.2 (4)	C32—C14—C27—O8	178.4 (3)
C14—C36—C47—C16	60.7 (5)	C24—C14—C27—O8	−61.7 (4)
C14—C36—C47—C51	−59.1 (4)	C36—C14—C27—O7	−124.1 (4)
C55—C34—C48—C22	177.8 (3)	C32—C14—C27—O7	−2.9 (5)
C46—C34—C48—C22	−58.3 (4)	C24—C14—C27—O7	117.0 (4)
C25—C34—C48—C22	60.3 (4)	C11—N2—C2—C10	1.4 (6)
C16—C47—C51—C21	−59.6 (4)	Zn1^ii^—N2—C2—C10	−177.5 (3)
C36—C47—C51—C21	60.1 (4)	C8—C10—C2—N2	0.9 (6)

Symmetry codes: (i) *x*−1/2, −*y*+1/2, *z*+1/2; (ii) *x*+1/2, −*y*+1/2, *z*−1/2.

### Hydrogen-bond geometry (Å, °)

**Table:**

*D*—H···*A*	*D*—H	H···*A*	*D*···*A*	*D*—H···*A*
O9—H9A···O1^iii^	0.82	2.05	2.872 (4)	178
O6—H6A···O3^iv^	0.82	2.00	2.812 (4)	169
O3—H3A···O9^v^	0.82	1.99	2.790 (4)	165
O1W—H1WA···O6	0.85	2.19	3.013 (9)	163
O5—H5C···O7	0.85	1.67	2.447 (3)	151
O1W—H1WB···O7^vi^	0.85	2.44	3.287 (9)	179

Symmetry codes: (iii) *x*+1/2, −*y*+1/2, *z*+1/2; (iv) *x*, −*y*+1, *z*−1/2; (v) *x*−1/2, *y*+1/2, *z*; (vi) *x*, −*y*, *z*−1/2.

## References

[bb1] Bruker (2006). *APEX2* and *SAINT* Bruker AXS Inc., Madison, Wisconsin, USA.

[bb2] Flack, H. D. (1983). *Acta Cryst.* A**39**, 876–881.

[bb3] Korlyukov, A. A., Komissarov, E. A. & Antipin, M. Y. (2008). *J. Mol. Struct.* **875**, 135–142.

[bb4] Milios, C. J., Inglis, R. & Bagai, R. (2007). *Chem. Commun.* **33**, 3476–3478. 10.1039/b705170k 17700887

[bb5] Sheldrick, G. M. (1996). *SADABS* University of Göttingen, Germany.

[bb6] Sheldrick, G. M. (2008). *Acta Cryst.* A**64**, 112–122. 10.1107/S0108767307043930 18156677

[bb7] Zhu, Z.-L., Feng, Y.-L., Lin, H. & Chin, J. (2005). *Rare Earth Soc.* **23**, 641–644.

